# The social determinants of depression: social support, loneliness, and the impact of the COVID-19 pandemic

**DOI:** 10.1192/j.eurpsy.2024.419

**Published:** 2024-08-27

**Authors:** A. Gabarrell-Pascuet, J. M. Haro, J. Domènech-Abella

**Affiliations:** Sant Joan de Déu Research Institute, Sant Boi de Llobregat, Spain

## Abstract

**Introduction:**

The COVID-19 pandemic involved stringent social restrictions, a surge in mortality, and significant economic consequences, affecting age groups differently and leading to increases in loneliness and mental health problems, particularly depression, which was already very common before the pandemic.

**Objectives:**

Analyse changes and related factors of the relationship between loneliness and depression by age group from (1) before to the COVID-19 outbreak, (2) during the pandemic, and (3) after the last state of emergency. Moreover, we aim to (4) evaluate the effect of social support to alleviate feelings of loneliness and improve the course of depression.

**Methods:**

We used data from three different cohorts, all representative of the Spanish adult population. (1) We longitudinally analysed the association between loneliness and depression with a sample interviewed before (N = 1,880) and during (N = 1,103) the pandemic. We used mixed-models to study changes in major depressive disorder (MDD) by age group and regression models to quantify the association between age and potential mediating effects. (2) We analysed data of 2,000 adults during the pandemic. Several regression models were constructed to assess the impact of pre-pandemic mental disorders on the main association by age group. (3) Out of those 2,000 participants, 1,300 were interviewed 9 months later, to determine group-based loneliness trajectories and its associated risk factors. (4) We analysed the relationship between loneliness, social support, and MDD over a 7-year period (N=404 individuals aged 50+ having MDD). We tested cross‐lagged panel models using structural equation modelling.

**Results:**

During the pandemic the probability of having MDD increased significantly among younger individuals, and was partly explained by loneliness, low resilience, and worsened economic situation. Loneliness was associated with more depressive symptoms, and this association was stronger in younger adults without pre-pandemic mental disorders and in older adults with them. At the end of pandemic, three courses of loneliness were detected: invariant low loneliness (42.6%), decreasing medium loneliness (51.5%), and fairly invariant high loneliness (5.9%). Risk factors for worse trajectories were being younger, female, not married, and, notably, having pre-pandemic mental disorders. Among individuals with depression prior to the pandemic, lower social support predicted higher subsequent levels of loneliness, resulting in an increase in MDD recurrence.

**Conclusions:**

Strategies to decrease the impact of loneliness on depressive symptoms should consider individuals mental health background, address social determinants, and adopt an age-driven perspective.

**Disclosure of Interest:**

None Declared

